# Influence of structured output constraints on GPT-5-Thinking, Gemini 2.5 Pro, and open-weight LLMs for radiology protocol selection

**DOI:** 10.1186/s41747-026-00712-3

**Published:** 2026-04-10

**Authors:** Mohammed Bahaaeldin, Sebastian Nowak, Oleksandra Seidel, Alexander Isaak, Dmitrij Kravchenko, Annemarie Proff, Tatjana Dell, Daniel Kuetting, Julian A. Luetkens, Narine Mesropyan

**Affiliations:** 1 Department of Diagnostic and Interventional Radiology, University Hospital Bonn, Bonn, Germany; 2 Quantitative Imaging Laboratory Bonn (QILaB), University Hospital Bonn, Bonn, Germany

**Keywords:** Constrained prompting, Large language models, Radiology report form, Protocol selection, Unconstrained prompting

## Abstract

**Objective:**

To evaluate the impact of constraining proprietary and open large language models (LLMs) to structured outputs in processing radiology request forms (RRF).

**Materials and methods:**

We evaluated five LLMs—two proprietary (GPT-5-Thinking, Gemini 2.5 Pro) and three open (Qwen3-235B-A22B-Thinking, gpt-oss-120b, medgemma-27b-it)—on 100 RRFs (50 computed tomography, 50 magnetic resonance imaging). Each model processed cases with and without constraints to structured outputs. Endpoints included accuracy for modality, anatomical region, contrast phase, urgency, “all correct” (all four categories correct), and “indication improved” (clarity of rewritten text). Outputs were evaluated against a reference standard defined by board-certified radiologists and compared with two radiology residents (first-year and third-year). Accuracies with 95% confidence intervals were calculated.

**Results:**

Constraining to structured outputs had model-dependent effects: it improved Gemini 2.5 Pro (all correct: from 53.0% [43.3–62.5] to 66.0% [56.3–74.5]) but reduced GPT-5-Thinking accuracy (from 76.0% [66.8–83.3] to 53.0% [43.3–62.5]), with minimal influence on open models. Both proprietary LLMs outperformed the best open models (up to 41.0% [31.9–50.8]). All LLMs exceeded the unassisted first-year residents’ performance (19.0% [12.5–27.8]). LLM assistance improved first-year residents’ accuracy to 65.0% [55.3–73.6], approaching the third-year residents’ performance (80.0% [71.1–86.7]), who performed comparably to the best LLMs. Across models, performance was highest for modality and anatomical region, and lowest for urgency. Indication reformulation was judged clearer in > 90% of cases across all models without hallucinations.

**Conclusion:**

Constraining to structured outputs exerted model-specific effects. Proprietary LLMs achieved the highest accuracy in RRF-based protocol selection and improved first-year resident performance to an experienced-resident level.

**Relevance statement:**

LLMs may serve as valuable decision-support tools for radiology workflow. Constraining LLMs to structured outputs produced divergent, model-specific effects in radiology protocol selection—improving Gemini 2.5 Pro, reducing GPT-5-Thinking, and minimally affecting open models—highlighting the need for model-specific prompting strategies before adopting LLMs in radiology decision support.

**Key Points:**

- Structured output constraints affect LLM performance differently.
- Gemini 2.5 Pro benefits from structured prompting, while GPT-5-Thinking declines.
- Open-weight models show minimal impact from output constraining.
- Proprietary models outperform open models in radiology protocol selection.

**Graphical Abstract:**

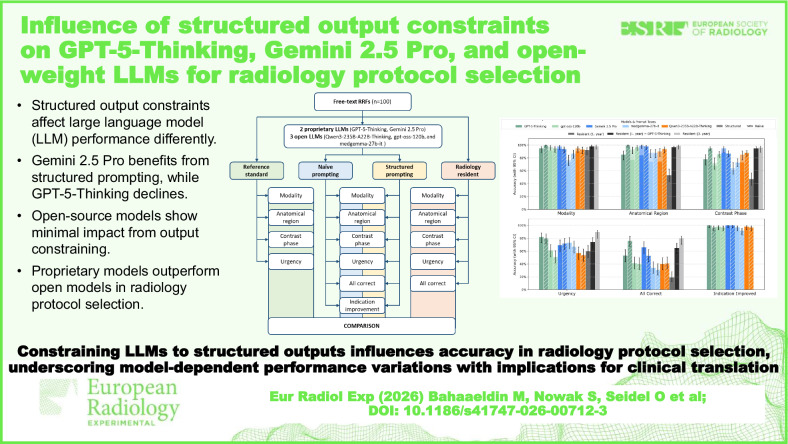

## Background

Cross-sectional imaging plays an increasingly important role in patient management, resulting in a continuous rise in examination volumes within radiology departments [1]. Processing radiology request forms (RRFs) represents a critical step in the radiology workflow. These forms are often brief, unstructured, and contain ambiguous abbreviations, making the selection of the most appropriate examination—including modality, anatomical region, contrast use, and urgency—resource-intensive for the responsible board-certified radiologists.

Large language models (LLMs) have attracted growing interest in radiology, with recent studies demonstrating their utility for tasks such as report generation, error detection, extraction of structured information from free-text reports, and clinical workflow support [1–11]. Extending these applications, a few recent studies have applied LLMs to the processing of RRFs, indicating their potential to enable consistent and accurate protocol selection [2, 3, 5, 12]. In general, two prompting strategies can be applied to LLMs, including for tasks such as protocol selection: (1) with constraining the model to structured outputs with predefined, machine-readable outputs, and (2) without any constraints to LLM output format (termed naive prompting). Prompting with predefined selections and output structures is essential for automated integration into clinical systems. While it may increase accuracy and reduce hallucinations, structured prompting can also restrict flexibility and lower performance. How such strategies affect LLM performance in radiology, including protocol selection from RRFs, remains unclear and has not yet been systematically evaluated.

Another important distinction concerns the two types of LLMs: proprietary frontier models (*e.g.,* GPT-5-Thinking, Gemini 2.5 Pro) and open models (*e.g.,* gpt-oss-120b, medgemma-27b-it, Qwen3-235B-A22B-Thinking) [13]. Proprietary models often achieve state-of-the-art performance. Although they can technically run on any sufficiently powerful hardware—including within secured clinical-network environments—current licensing and deployment constraints typically require execution on external company servers. This raises data protection and governance concerns, whereas the licensing and deployment requirements of open-weight models typically allow local deployment on institution-managed hardware and operation within secure clinical networks. For sites prioritizing data locality and system control, on-premise open models are therefore attractive—provided their outputs are reliable and readily integrable.

Therefore, the aim of this exploratory study was to investigate the influence of prompting strategies on LLMs for protocol selection based on RRFs, and to systematically compare state-of-the-art proprietary (GPT-5-Thinking, Gemini 2.5 Pro) and open models (gpt-oss-120b, medgemma-27b-it, Qwen3-235B-A22B-Thinking). Their performance (evaluated for modality choice, anatomical region, contrast phase, urgency, and indication quality) was benchmarked against an expert radiologist consensus and a radiology resident to assess their potential utility as clinical decision-support tools.

## Materials and methods

### Study design and data

A total of 100 fully anonymized RRFs were randomly extracted from the local Radiology Information System using stratified sampling to reflect the institutional subspecialty distribution. Inspired by these real clinical cases, 100 RRFs were generated by an independent radiologist blinded to the study objectives. Real-world characteristics—including telegraphic style, standard and non-standard abbreviations, typographical errors, under specification, and ambiguity—were intentionally preserved to authentically recreate the complexity of routine clinical practice. No patient or patient-identifying information was included or provided to LLMs.

### **LLMs and experiments**

**Fig. 1:**
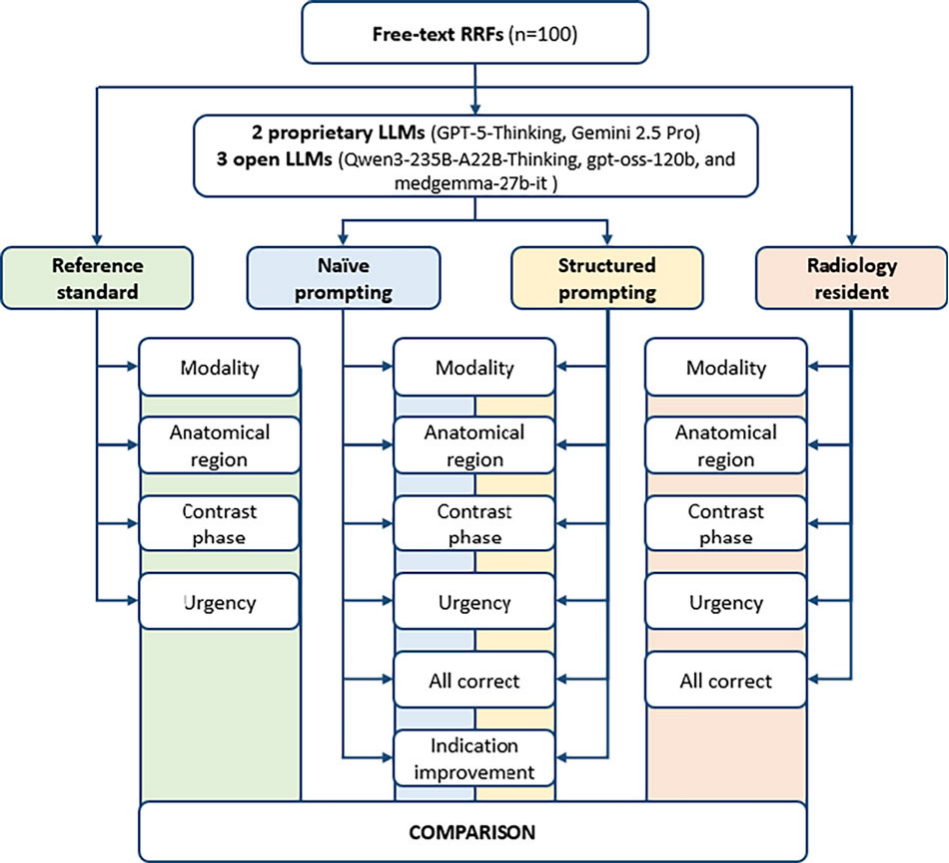
Study design. A total of 100 anonymized free-text RRFs were evaluated. Five LLMs—two proprietary (GPT-5-Thinking, Gemini 2.5 Pro) and three open (Qwen3-235B-A22B-Thinking, gpt-oss-120b, medgemma-27b-it)—processed each case under both naïve and structured prompting conditions. Model outputs were compared against a reference standard established by two board-certified radiologists and against the performance of two radiology residents (a first-year and a third-year resident). Evaluation endpoints included modality, anatomical region, contrast phase, urgency, “all correct,” and indication improvement. LLMs, Large language models; RRFs, Radiology request forms

A flow diagram summarizing the study design is shown in Fig. 1. Five of the most advanced LLMs were evaluated:

1. two proprietary models: GPT-5-Thinking (OpenAI) and Gemini 2.5 Pro (Google DeepMind);
2. three open models: gpt-oss-120b (OpenAI), medgemma-27b-it (Google DeepMind), and Qwen3-235B-A22B-Thinking (Alibaba Group).

Each model was tested using two distinct prompting strategies: prompting with constraints for output structure and naïve, unconstrained prompting.

### Prompting with constraints

Models were requested to determine the appropriate cross-sectional imaging scan properties as JavaScript Object Notation‒JSON output following a strict schema with predefined selection possibilities (Appendix S1). This schema included modality (single selection of computed tomography [CT] or magnetic resonance imaging [MRI]), anatomical regions (multiple selection of 31 predefined regions), contrast strategy (allowed multiple selection of native, arterial, portal venous, late venous, urographic-phase), and examination priority (routine [> 3 days], urgent [1–3 days], or immediate [≤ 24 h]). For the open models, we applied grammar guided-structured decoding [14]. Also, the model was required to generate an improved anamnesis text and description of the clinical question based on the provided, short RRFs.

The results of constrained prompting underwent a two-stage evaluation. First, the structured fields were automatically compared against a reference standard established by two radiologists in consensus (N.M. and J.A.L.). Exact matches were considered correct. Second, disagreements were inspected and classified as acceptable or unacceptable differences by the radiologists, except for classifications regarding the urgency of the examination, which were always considered incorrect in case of a mismatch. In addition, the “all correct” category was evaluated manually by the same two radiologists, defined as cases in which an LLM matched the expert reference across all categories simultaneously (modality, anatomical region, contrast phase, and urgency). For the “indication improved” category, a radiologist reviewed the LLM-generated text and rated it as “improved” or “not improved,” based on whether it was clearer and more clinically precise than the original, while ensuring that no factual inaccuracies or hallucinations had been introduced. A detailed description of the prompts is provided in Supplemental material, Section S1.

### Naïve, unconstrained prompting

For naïve prompting, no constraints regarding output structure or predefined categories for anatomical regions or contrast strategy were introduced to the LLMs. So the first evaluation stage—automatic comparison to the structured reference standard—was not applicable. Therefore, all outputs were manually reviewed and classified by the radiologists according to the second-stage evaluation procedure used for constraint prompting.

The closed models were accessed via their respective web interface platforms by M.B., with exclusion of data used for future training. The open models were deployed using LLM on a local 8× A100 80GB compute node using vLLM by S.N. [15]. The reference standard was established by two experienced board-certified radiologists (N.M., 7 years of experience; J.A.L., 15 years of experience) who assigned each category in consensus.

LLM performance was compared with that of two radiology residents: an inexperienced first-year resident (O.S.) and an experienced third-year resident (A.P.). Additionally, the same first-year radiology resident repeated the rating task under otherwise identical conditions, using GPT-5 Thinking (naïve) as a decision-support tool and applying the same rating approach as in the initial experiment.

### **Statistical analysis**

Statistical analysis was performed using Python v3.11 and its scientific computing libraries, including pandas for data management, NumPy v2.2.6 for numerical operations, and SciPy v1.16.0 for statistical functions. Accuracy was the primary performance metric, calculated for each model and prompt type across all evaluation categories. The 95% confidence intervals (CIs) for accuracy were calculated using the Wilson score interval method. We define relevant differences in performance between models or conditions as those where the 95% CIs do not overlap.

## Results

### Study sample

The dataset comprised 100 RRFs (50 CT and 50 MRI), distributed across inflammatory (*n* = 9), cardiovascular (*n* = 21), gastrointestinal (*n* = 9), musculoskeletal (*n* = 13), neurologic (*n* = 5), oncologic (*n* = 31), and trauma/emergency (*n* = 12) cases. Controlled vocabularies included the full body-region list and the contrast phases: native, arterial, portal venous, late venous, and urographic.

### Impact of prompting approach on model performance

**Fig. 2:**
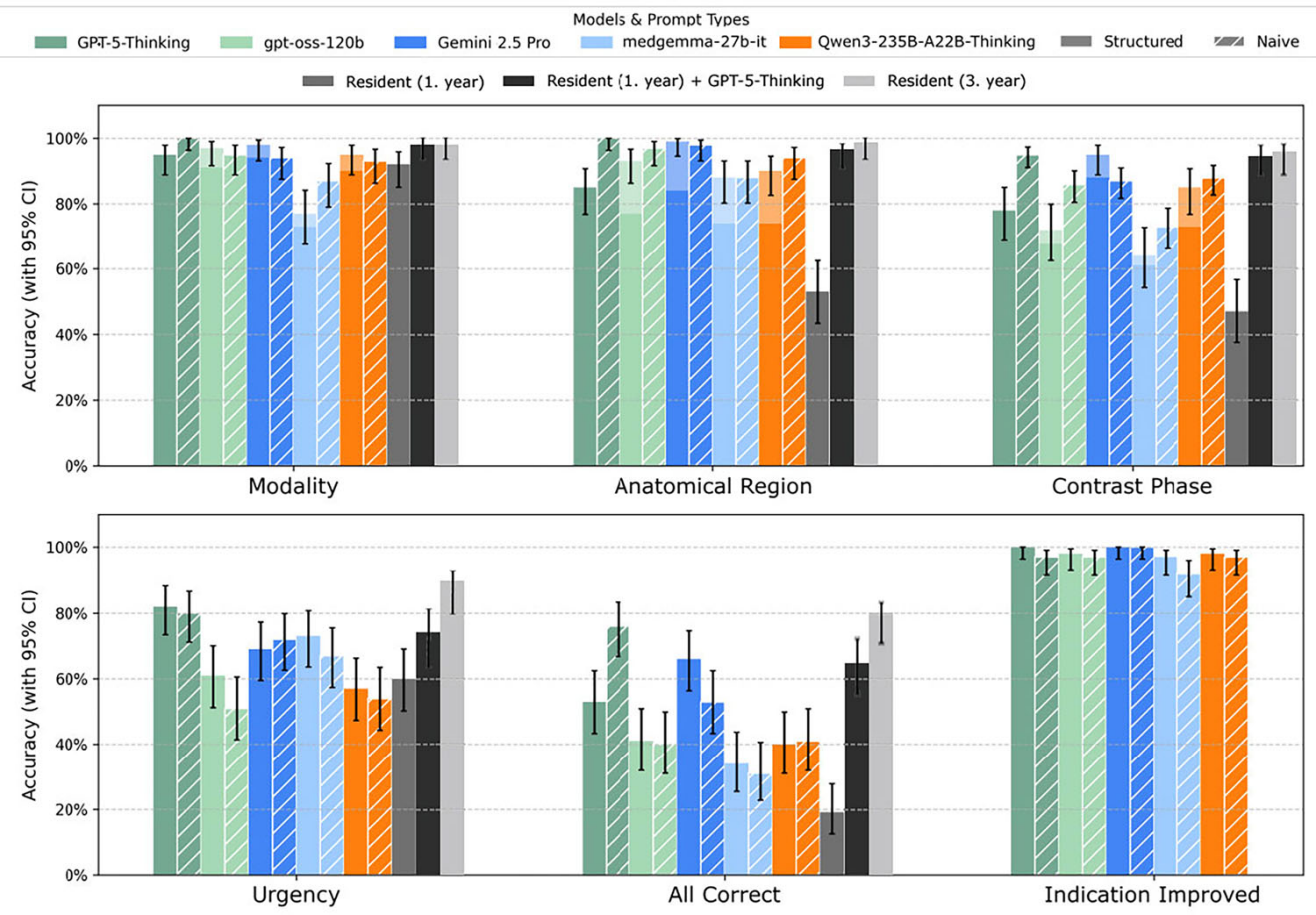
Accuracy of LLMs by prompting approach and endpoint. Bar charts show accuracy (%) with 95% CIs for all tested models under naïve and structured prompting, as well as for both radiology residents (first-year; third-year), compared with the reference standard. In addition, the performance of the first-year radiology resident assisted by GPT-5 Thinking (naïve) was evaluated. Results are presented across six endpoints: modality, anatomical region, and contrast phase (top row); urgency, “all correct,” and “indication improved” (bottom row). “All correct” indicates that all four categories (modality, anatomical region, contrast phase, and urgency) were correctly assigned for a given case, whereas “indication improved” reflects a radiologist’s judgment that the LLM-reformulated indication was clearer than the original request form without factual alteration. CI, Confidence intervals; LLMs, Large language models

The choice of prompting strategy had a model-dependent impact (Table 1 and Fig. 2). Among proprietary LLMs, constraining to structured outputs yielded opposite effects: for GPT-5-Thinking, “all correct” accuracy relevantly decreased compared with naïve prompting (53.0% [43.3–62.5] *versus* 76.0% [66.8–83.3]). By contrast, Gemini 2.5 Pro improved by constraining to structured outputs (66.0% [56.3–74.5] *versus* 53.0% [43.3–62.5]). For open models (gpt-oss-120b, Qwen3-235B-A22B-Thinking, medgemma-27b-it), structured prompting had minor and non-relevant effects on performance. For example, gpt-oss-120b achieved nearly identical accuracies for “all correct” assignment under naïve and structured conditions (40.0% [30.9–49.8] *versus* 41.0% [31.9–50.8]), and Qwen3-235B-A22B-Thinking showed a similar pattern (41.0% [31.9–50.8] *versus* 40.0% [30.9–49.8]). All accuracies with corresponding CIs for all LLMs under naïve and structured conditions are summarized in Table 1 and illustrated in Fig. 2.

**Table 1:** Summary of overall accuracy for determining imaging protocol (modality, anatomical region, contrast phase, urgency) based on RRFs by LLMs and two radiology residents (a first-year and a third-year resident) in comparison with the reference standard

Model (prompt)	Modality (%)	Anatomical region (%)	Contrast phase (%)	Urgency (%)	All correct (%)	Indication improved (%)
GPT-5-Thinking (naïve)	100.0 [96.3‒100.0]	100.0 [96.3‒100.0]	95.0 [91.0‒97.3]	80.0 [71.1‒86.7]	76.0 [66.8‒83.3]	97.0 [91.5‒99.0]
GPT-5-Thinking (structured)	95.0 [88.8‒97.8]	85.0 [76.7‒90.7]	78.0 [68.9‒85.0]	82.0 [73.3‒88.3]	53.0 [43.3‒62.5]	100.0 [96.3‒100.0]
Gemini-2.5 Pro (naïve)	94.0 [87.5‒97.2]	98.0 [93.0‒99.4]	87.0 [81.6‒91.0]	72.0 [62.5‒79.9]	53.0 [43.3‒62.5]	100.0 [96.3‒100.0]
Gemini-2.5 Pro (structured)	98.0 [93.0‒99.4]	99.0 [94.6‒99.8]	95.0 [88.8‒97.8]	69.0 [59.4‒77.2]	66.0 [56.3‒74.5]	100.0 [96.3‒100.0]
Qwen3-235B-A22B-Thinking (naïve)	93.0 [86.3‒96.6]	94.0 [87.5‒97.2]	87.9 [82.7‒91.8]	54.0 [44.3‒63.4]	41.0 [31.9‒50.8]	97.0 [91.5‒99.0]
Qwen3-235B-A22B-Thinking (structured)	95.0 [88.8‒97.8]	90.0 [82.6‒94.5]	85.0 [76.7‒90.7]	57.0 [47.2‒66.3]	40.0 [30.9‒49.8]	98.0 [93.0‒99.4]
gpt-oss-120b (naïve)	95.0 [88.8‒97.8]	97.0 [91.5‒99.0]	85.9 [80.4‒90.1]	51.0 [41.3‒60.6]	40.0 [30.9‒49.8]	97.0 [91.5‒99.0]
gpt-oss-120b (structured)	97.0 [91.5‒99.0]	93.0 [86.3‒96.6]	72.0 [62.5‒79.9]	61.0 [51.2‒70.0]	41.0 [31.9‒50.8]	98.0 [93.0‒99.4]
medgemma-27b-it (naïve)	87.0 [79.0‒92.2]	88.0 [80.2‒93.0]	73.0 [66.5‒78.7]	67.0 [57.3‒75.4]	31.0 [22.8‒40.6]	92.0 [85.0‒95.9]
medgemma-27b-it (structured)	77.0 [67.8‒84.2]	88.0 [80.2‒93.0]	64.0 [54.2‒72.7]	73.0 [63.6‒80.7]	34.0 [25.5‒43.7]	97.0 [91.5‒99.0]
First-year radiology resident	92.0 [85.0‒95.9]	53.0 [43.3‒62.5]	47.0 [37.5‒56.7]	60.0 [50.2‒69.1]	19.0 [12.5‒27.8]	‒
First-year radiology resident assisted by GPT-5 (naïve)	98.0% [93.0‒99.4]	96.0% [90.2‒98.4]	94.0% [87.5‒97.2]	74.0% [64.6‒81.6]	65.0% [55.3‒73.6]	‒
Third-year radiology resident	98.0% [93.0‒99.4	98.0% [93.0‒99.4]	95.0% [88.8‒97.8]	88.0% [80.2‒93.0]	80.0% [71.1‒86.7]	‒

Values are reported as accuracy in percent with 95% CIs in parentheses. ”Indication Improved” shows the percentage of RRFs that were considered as improved by the LLMs

### **Performance of proprietary*versus*open-weight models**

All models generally performed well in determining modality and anatomical region, with mostly minimal differences between open and proprietary LLMs; this performance gap narrowed further under constraints to structured outputs (Fig. 2 and Table 1).

Similarly, contrast phase assignment showed non-relevant difference between proprietary and open models, with the best proprietary model being GPT-5-Thinking (naïve: 95.0 [91.0–97.3]) and best open being Qwen3-235B-A22B-Thinking (naïve: 87.9 [82.7–91.8]). In contrast, urgency was the most challenging category for all LLMs, with no relevant difference between proprietary and open models, with the best proprietary model being GPT-5-Thinking (structured: 82.0% [73.3–88.3]) and best open model being medgemma-27b-it (73.0 [63.6–80.7]). In the “all correct” category, proprietary LLMs performed significantly better than open models. GPT-5-Thinking reached the highest accuracy (naive: 76.0% [66.8–83.3]), which has a relevant difference to the best results yielded by open models, which were for structured GPT-oss-120 b and naive Qwen3-235B-A22B-Thinking (41.0% [31.9–50.8]).

### LLM's performance on indication improvement

All models performed exceptionally well in reformulating the original indication text into a clearer and more structured format (Fig. 2 and Table 1). As shown in the “Indication Improved” panel of Fig. 1, accuracies across all models and prompting strategies clustered near 100%, demonstrating a consistent and robust ability to enhance the linguistic quality of clinical texts without hallucinations.

### **Clinical implication: performance of LLMs*versus*radiology residents**

Across most evaluation categories, all LLMs outperformed the first-year radiology resident, except for modality and urgency assignment, where performances were largely comparable. The largest differences emerged in the “all correct” category: accuracy was 19.0% (95% CI: 12.5–27.8) for the first-year radiology resident, which was significantly lower than all LLMs, except medgemma-27b-it. However, when assisted by GPT-5 Thinking (naïve), the first-year radiology resident’s performance improved across all categories, with the largest gain observed in the “all correct” category (19.0% [95% CI: 12.5–27.8] to 65.0% [95% CI: 55.3–73.6]).

The more experienced third-year radiology resident performed comparably to GPT-5 Thinking (naïve) and even slightly outperformed it in the “urgency” (88.0% [95% CI: 80.2–93.0] *versus* 80.0% [95% CI: 71.1–86.7]) and “all correct” (80.0% [95% CI: 71.1–86.7] *versus* 76.0% [95% CI: 66.8–83.3]) categories.

## Discussion

In this exploratory study, we investigated the influence of constraining LLMs to structured outputs when determining appropriate imaging protocols from RRFs. We systematically compared state-of-the art proprietary (GPT-5-Thinking, Gemini 2.5 Pro) and open models (gpt-oss-120b, medgemma-27b, Qwen3-235B-A22B-Thinking). Performance across modality, anatomical region, contrast phase, urgency, and indication quality was benchmarked against an expert consensus and compared with that of a radiology resident. The main findings of our study are: (1) constraining frontier LLMs to structured outputs had model-dependent effects, with structured prompting improving Gemini 2.5 Pro (“all correct“ accuracy 53.0% [43.3–62.5] → 66.0% [56.3–74.5]) but reducing GPT-5-Thinking performance (76.0% [66.8–83.3] → 53.0% [43.3–62.5]), while effects on open models were minimal; (2) proprietary LLMs, particularly GPT-5-Thinking, achieved the highest overall accuracy in RRF processing compared with open models (“all correct“ accuracy up to 76.0% [66.8–83.3] for GPT-5-Thinking *versus* 41.0% [31.9–50.8] for Qwen3-235B-A22B-Thinking and gpt-oss-120b); and (3) all LLMs achieved higher concordance with board-certified radiologists in protocol selection than the radiology resident. (*e.g.,* “all correct“ accuracy GPT-5-Thinking 76.0% [66.8–83.3] *versus* 19.0% [12.5–27.8]).

LLMs are gaining increasing attention in medicine, with applications in clinical decision support and workflow optimization [4, 6]. In radiology, recent studies have demonstrated their utility for automated report generation, error and inconsistency detection, extraction of structured findings from free-text reports, and integration into clinical workflow systems [4, 5, 7–10]. Several recent studies have also applied LLMs to protocol selection based on RRFs, highlighting their potential to streamline this critical step in clinical practice [2, 3, 5, 10, 12]. Gertz et al showed that GPT-4 correctly selected modality and protocol in ~84% of real request forms [5], while Zaki et al reported that a domain-trained model (Glass AI) outperformed ChatGPT across more than 1,000 ACR-based scenarios [10]. Another recent study demonstrated that advanced models such as DeepSeek-R1 achieved accuracies up to 98% and matched or exceeded radiology resident appropriateness ratings [12]. Beyond exam selection, GPT-4 showed that it could generate MRI protocols with completeness and utility comparable to a radiology resident, with 95% deemed clinically applicable [3]. Overall, these findings indicate that LLMs can support modality choice, protocol generation, and guideline-based appropriateness at a performance level approaching or exceeding that of trainees, while offering substantially improved efficiency [3–10, 12]. To our knowledge, the influence of structured output constraints on the performance of current proprietary and open models in radiology has not yet been systematically evaluated.

In our study, structured prompting showed model-dependent effects. For GPT-5-Thinking, performance decreased under rigid schema constraints, suggesting that highly advanced models may be hindered by “box-ticking” formats that restrict nuanced reasoning and reduce accuracy in complex cases. By contrast, Gemini benefited from structured prompting, with improvements across nearly all categories—including the “all correct” measure—indicating that added structure reduced variability and anchored decisions to standardized categories. More generally, unconstrained prompts allow models to generate flexible outputs, but the output structure varies between runs. This structural inconsistency limits reproducibility and prevents direct integration into databases or automated clinical systems [14]. This variability poses challenges for downstream applications such as automated scanner scheduling, which require standardized and machine-readable outputs. Constraining models to predefined categories and formats (*e.g.,* JavaScript Object Notation‒JSON) addresses this need, enabling seamless workflow integration, but at the potential cost of reduced flexibility. These findings highlight that structured prompting is neither universally beneficial nor detrimental; rather, its impact reflects a trade-off between flexibility, accuracy, and interoperability, depending on the model and clinical context. However, this does not render unconstrained outputs clinically unusable. In a practical deployment scenario, a two-step workflow could be implemented: the model first generates an unconstrained output, which is then converted into a structured format via a second prompt. This approach would combine the strong reasoning performance observed with naïve prompting (*e.g.,* GPT-5 Thinking) with the structured output required for Radiology Information System integration.

When comparing proprietary and open LLMs, we found that open models performed largely competitively with proprietary models in individual endpoints such as modality and anatomical region. However, a clear divergence in performance was observed between technical protocol selection and exam prioritization. Urgency posed the most challenging endpoint for all LLMs, likely reflecting the limited urgency cues in short RRFs and frequent misclassification of non-urgent cases (*e.g.,* preoperative planning) as “red flags.” The performance gap between proprietary and open models narrowed under structured prompting, but proprietary models—particularly GPT-5-Thinking—continued to achieve the highest accuracy in naïve settings. Beyond accuracy, proprietary frontier models raise substantial data privacy and governance concerns, as licensing and deployment constraints typically require processing on external company servers. Although regulatory requirements could theoretically be met through extensive server security certification and individual liability agreements, processing protected health information remains practically near-impossible—particularly for EU institutions—because model providers do not currently offer such agreements at scale. [4, 6, 10]. These limitations pose significant barriers to direct clinical integration, even when performance is superior. By contrast, open models can be deployed locally within secure clinical networks and are more readily integrated into existing hospital workflows.

Another important finding is that all LLMs, independent of prompting strategy or model type, outperformed the first-year radiology resident in RRF processing, underscoring their potential as clinical decision-support tools. This was further supported by an additional experiment in which the same first-year radiology resident was assisted by GPT-5 Thinking (naïve). This is in line with previous studies [2, 3, 12]. By transforming brief and ambiguous referral notes into standardized indications, LLMs can improve communication between referring physicians and radiologists, reduce errors and unnecessary clarifications, and facilitate the selection of appropriate imaging protocols. Clinically, this suggests that LLM-based decision support may be most beneficial for junior residents—particularly in their first year—thereby supporting less experienced radiologists and improving overall workflow efficiency.

This study has several limitations. First, although the RRFs were designed to reflect real-world request forms, they were synthetically rewritten. While this ensured complete anonymization, it may not fully capture the diversity, complexity, and variability of authentic clinical requests, potentially limiting generalizability. Second, the study was conducted with RRFs from a single institution, which may introduce institutional bias related to subspecialty distribution, terminology, and referral practices. Third, only 100 RRFs (50 CT and 50 MRI) were included. Although stratified sampling was applied, the sample size remains limited relative to the broad spectrum of indications encountered in daily practice. Fourth, performance was assessed only in predefined categories (modality, anatomical region, contrast strategy, urgency, and reformulation of indication text). Other clinically relevant tasks—such as suggesting imaging alternatives, handling incomplete or contradictory information, or integrating patient history—were not evaluated. Further, the study focused on offline evaluation of RRFs. Real-world aspects such as Radiology Information System/ Picture Archiving and Communication System integration, response time, user interaction, and downstream effects on workflow and patient care were not assessed. In addition, as this study was designed as an exploratory generation of data rather than a hypothesis-driven trial, we did not assess statistical significance for inter-model differences, as the extensive corrections required for multiple testing would have limited the insightfulness of the analysis. Therefore, these exploratory findings should be viewed as descriptive and validated in future studies with dedicated power analyses. Furthermore, as a time interval occurred between the first-year resident’s initial and secondary evaluations, the observed performance improvement with artificial intelligence (AI) assistance could partially be attributed to the resident’s clinical experience gained during this period rather than the model alone. Finally, while larger open-weight models generally exhibited higher performance, a comprehensive correlation analysis was beyond the scope of this work and was also not feasible due to undisclosed parameter counts for closed-weight models. Nonetheless, our primary focus was on the intra-model impact of structured prompting rather than on confirming established scaling laws [16]. To further improve the performance of privacy-preserving local models beyond prompting strategies, future work should investigate advanced optimization techniques such as retrieval-augmented generation or fine-tuning via low-rank adaptation [7]. Therefore, further multicenter studies with larger cohorts are warranted to address these limitations, while carefully considering data privacy policies and governance aspects.

In conclusion, this study reveals a complex and model-dependent relationship between prompting strategy and performance in the task of processing RRFs across the most advanced existing open and proprietary LLMs.

## Supplementary information

**Appendix S1** Naïve and structured prompts provided to the LLMs for RRF processing

## Data Availability

The datasets used and/or analysed during the current study are available from the corresponding author on reasonable request.

## Abbreviations

CI: Confidence interval
CT: Computed tomography
LLM: Large language model
MRI: Magnetic resonance imaging
RRF: Radiology request form
